# P-380. The Clinical Development of VH3810109 (N6LS): Advancing Ultra-Long-Acting HIV Treatment into the Future

**DOI:** 10.1093/ofid/ofaf695.598

**Published:** 2026-01-11

**Authors:** Peter Leone, Paul Wannamaker, Margaret Gartland, Michael Warwick-Sanders, Riccardo D’Agostino, Rulan Griesel, Chelsea Macfarlane, Viviana Wilches, Kathryn Brown, Yash Gandhi, David Dorey, Gabriela L Ghita, Christina Donatti, Babafemi Taiwo, Sherene Min, Jan Losos

**Affiliations:** ViiV Healthcare, Chapel hill, North Carolina; ViiV Healthcare, Chapel hill, North Carolina; ViiV Healthcare, Chapel hill, North Carolina; GSK, London, England, United Kingdom; GSK, London, England, United Kingdom; ViiV Healthcare, Chapel hill, North Carolina; ViiV Healthcare, Chapel hill, North Carolina; GlaxoSmithKline, Collegeville, Pennsylvania; Certara, Radnor, Pennsylvania; GSK, London, England, United Kingdom; GSK, London, England, United Kingdom; GSK, London, England, United Kingdom; ViiV Healthcare, Chapel hill, North Carolina; ViiV Healthcare, Chapel hill, North Carolina; ViiV Healthcare, Chapel hill, North Carolina; ViiV Healthcare, Chapel hill, North Carolina

## Abstract

**Background:**

Advancing novel ultra-long-acting (ULA; ≥ 4-month dosing interval) antiretroviral therapies is imperative to provide more options, enhancing quality of life and adherence. Broadly neutralizing antibodies, such as N6LS, have been engineered to have long half-lives and are currently being investigated to provide ULA antiretroviral options. N6LS binds to the CD4-binding site of the HIV-1 envelope and prevents entry into the host target cell. N6LS showed broad neutralization activity in vitro and potent antiviral activity in preclinical studies. Here, we summarize findings from N6LS clinical studies that informed the clinical development program.

**Figure:**
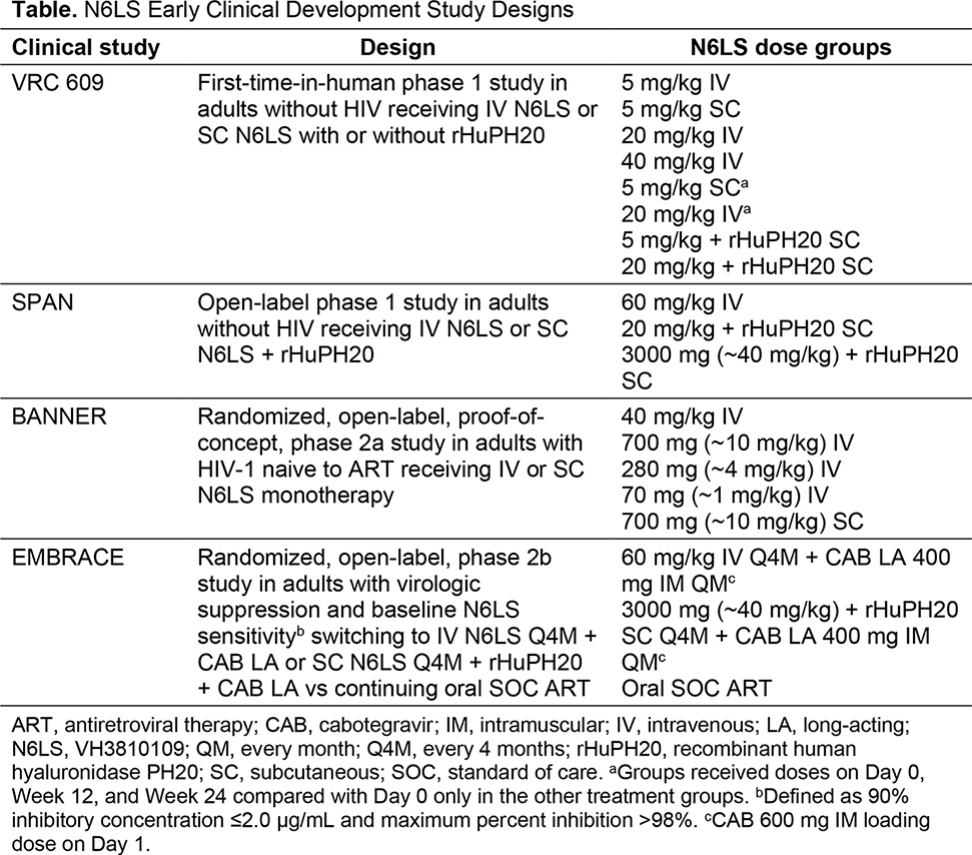

**Methods:**

The phase 1 VRC 609 (first-time-in-human) and SPAN studies evaluated pharmacokinetics and safety of intravenous (IV) N6LS and subcutaneous (SC) N6LS with or without recombinant human hyaluronidase PH20 (rHuPH20) in adults without HIV (Table). The proof-of-concept phase 2a BANNER study investigated antiviral activity and safety of IV or SC N6LS monotherapy in adults with HIV-1 naive to antiretroviral therapy (ART). The phase 2b EMBRACE study evaluated efficacy and safety of every-4-month (Q4M) N6LS (using doses from SPAN) + monthly long-acting cabotegravir (CAB LA) as a complete long-acting regimen in adults with HIV-1 and virologic suppression.

**Results:**

VRC 609 confirmed N6LS has a long half-life (40-44 days). SPAN indicated that N6LS could be safely administered 60 mg/kg IV or 3000 mg SC with rHuPH20. N6LS monotherapy showed robust antiviral activity in people naive to ART in BANNER, with ≥ 83% of participants receiving higher IV doses achieving virologic response (reduction in HIV-1 RNA ≥ 0.5 log_10_ copies/mL from baseline). Virologic suppression at 6 months was maintained in participants with baseline N6LS sensitivity receiving IV or SC N6LS Q4M + CAB LA in EMBRACE (96% and 88%, respectively). Both IV and SC N6LS were generally well tolerated across clinical studies, with low rates of infusion site reactions in the IV group, and a desirable safety profile.

**Conclusion:**

The N6LS development program aims to expand safe and effective ULA treatment options. Data from VRC 609, SPAN, and BANNER supported a Q4M evaluation of N6LS in EMBRACE; favorable results from these studies warrant the evaluation of twice-yearly N6LS.

**Disclosures:**

Peter Leone, MD, GSK: Stocks/Bonds (Public Company)|ViiV Healthcare: Employee Paul Wannamaker, BA, GSK: Stocks/Bonds (Public Company)|ViiV Healthcare: Employee Margaret Gartland, MSc, GSK: Stocks/Bonds (Public Company)|ViiV Healthcare: Employee Michael Warwick-Sanders, BM BSc DPM MFPM, GSK: Employee|GSK: Stocks/Bonds (Public Company) Riccardo D’Agostino, PhD, GSK: Employee|GSK: Stocks/Bonds (Public Company) Rulan Griesel, MBChB, GSK: Stocks/Bonds (Public Company)|ViiV Healthcare: Employee Chelsea Macfarlane, PhD, GSK: Stocks/Bonds (Public Company)|ViiV Healthcare: Employee Viviana Wilches, HBSc, MBiotech, GSK: Employee|GSK: Stocks/Bonds (Public Company) Kathryn Brown, PhD, Certara: Employee Yash Gandhi, PhD, GSK: Employee|GSK: Stocks/Bonds (Public Company) David Dorey, MMATH, GSK: Employee Gabriela L. Ghita, PhD, MPH, GSK: Employee|GSK: Stocks/Bonds (Public Company) Christina Donatti, PsyD, GSK: Stocks/Bonds (Public Company)|ViiV Healthcare: Employee Babafemi Taiwo, MBBS, GSK: Stocks/Bonds (Public Company)|ViiV Healthcare: Employee Sherene Min, MD, GSK: Stocks/Bonds (Public Company)|ViiV Healthcare: Employee Jan Losos, PhD, GSK: Stocks/Bonds (Public Company)|ViiV Healthcare: Employee

